# Effect of 23-Valent Pneumococcal Polysaccharide Vaccine Inoculated During Anti-Cancer Treatment Period in Elderly Lung Cancer Patients on Community-Acquired Pneumonia Hospitalization: A Nationwide Population-Based Cohort Study: Erratum

**DOI:** 10.1097/01.md.0000470159.45391.8f

**Published:** 2015-07-24

In the article “Effect of 23-Valent Pneumococcal Polysaccharide Vaccine Inoculated During Anti-Cancer Treatment Period in Elderly Lung Cancer Patients on Community-Acquired Pneumonia Hospitalization: A Nationwide Population-Based Cohort Study”,^1^ which appeared in Volume 94, Issue 26 of *Medicine*, an incorrect version of Figure 1 appeared. The article has since been corrected online.
